# Complete genome sequence of *Thauera aminoaromatica* strain MZ1T

**DOI:** 10.4056/sigs.2696029

**Published:** 2012-07-20

**Authors:** Ke Jiang, John Sanseverino, Archana Chauhan, Susan Lucas, Alex Copeland, Alla Lapidus, Tijana Glavina Del Rio, Eileen Dalin, Hope Tice, David Bruce, Lynne Goodwin, Sam Pitluck, David Sims, Thomas Brettin, John C. Detter, Cliff Han, Y.J. Chang, Frank Larimer, Miriam Land, Loren Hauser, Nikos C. Kyrpides, Natalia Mikhailova, Scott Moser, Patricia Jegier, Dan Close, Jennifer M. DeBruyn, Ying Wang, Alice C. Layton, Michael S. Allen, Gary S. Sayler

**Affiliations:** 1 Center for Environmental Biotechnology, The University of Tennessee, Knoxville, Tennessee, USA; 2 DOE Joint Genome Institute, Walnut Creek, California, USA; 3 Los Alamos National Laboratory, Bioscience Division, Los Alamos, New Mexico, USA; 4 Oak Ridge National Laboratory, Oak Ridge, Tennessee, USA; 5 Department of Biosystems Engineering and Soil Science, The University of Tennessee, Knoxville, Tennessee, USA; 6 Department of Biological Sciences, University of North Texas, Denton, Texas, USA

**Keywords:** *Thauera aminoaromatica*, MZ1T, genome

## Abstract

*Thauera aminoaromatica* strain MZ1T, an isolate belonging to genus *Thauera*, of the family *Rhodocyclaceae* and the class the *Betaproteobacteria*, has been characterized for its ability to produce abundant exopolysaccharide and degrade various aromatic compounds with nitrate as an electron acceptor. These properties, if fully understood at the genome-sequence level, can aid in environmental processing of organic matter in anaerobic cycles by short-circuiting a central anaerobic metabolite, acetate, from microbiological conversion to methane, a critical greenhouse gas. Strain MZ1T is the first strain from the genus *Thauera* with a completely sequenced genome. The 4,496,212 bp chromosome and 78,374 bp plasmid contain 4,071 protein-coding and 71 RNA genes, and were sequenced as part of the DOE Community Sequencing Program CSP_776774.

## Introduction

Strain MZ1T (=DSM 25461 =MTCC 11151=LMG 26735), a Gram-negative bacterium, was isolated from activated sludge samples from the industrial wastewater treatment facility of Eastman Chemical Company, Kingsport, Tennessee [1]. It is related to the genera *Azoarcus* and another prominent community member of activated sludge, *Zoogloea*. Strain MZ1T was identified as a significant component of microbial clusters formed during viscous bulking that resulted in poor sludge dewaterability and increased costs for dewatering, incineration and disposal [2]. Subsequently, MZ1T was found to produce a novel exopolysaccharide which contributed to the viscous bulking phenomenon. The genus *Thauera* is named after the German microbiologist Rudolf Thauer and was described by Macy et al. [3]. Currently, this genus consists of nine species with validly published names. These species have been isolated from a wide range of environments including wastewater activated sludge, water and soil, and typically degrade aromatic compounds such as benzoic acid or toluene under anaerobic conditions [3-8]. Here we present a summary classification and a set of features for *T. aminoaromatica* MZ1T, along with the description of the complete genomic sequencing and annotation.

## Classification and features

**Figure 1:**
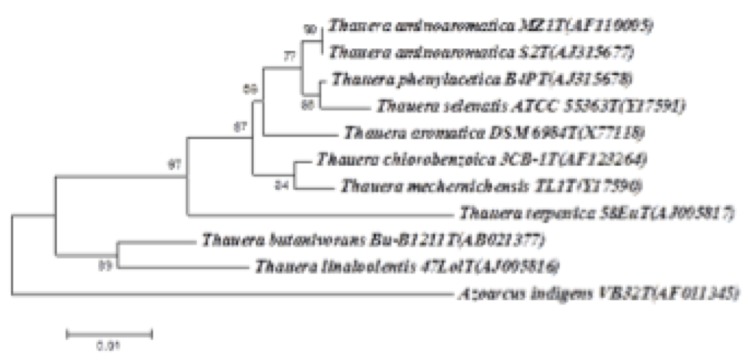
16S rDNA based phylogenetic tree depicting the relationship between *Thauera aminoaromatica* MZ1T and other members of the genus *Thauera*. The tree was constructed by using the Neighbor-Joining method and Jukes & Cantor evolutionary distance matrix from aligned 16S rDNA gene sequences and rooted using *Azoarcus indigens* as the outgroup. Bootstrap values (expressed as percentage of 500 replications) greater than 50 % are shown at the branch points. The branches are scaled as the number of base substitutions per site.

Strain MZ1T originally was identified as belonging to *Thauera* genus based on the 16S rRNA phylogenetic analysis [1].The sequences of the four 16S rRNA gene copies in the genome do not differ from each other. However, they differ from the previously published 16S rRNA sequence (AF110005), which contains one gap and eleven ambiguous base calls. Figure 1 shows the phylogenetic relationship of *T. aminoaromatica* MZ1T in a 16S rRNA based tree to other *Thauera* species. Based on this tree, strain MZ1T is closely grouped with *T. aminoaromatica* S2, *T. phenylacetica* B4P and *T. selenatis* and the cluster of these four strains is well-separated from strains of *T. aromatica, T. chlorobenzoica, T. mechernichensis, T. terpenica, T. butanivorans* and *T. linaloolentis*.

DNA-DNA hybridization was performed between strain MZ1T and *T. selenatis* ATCC 55363, *T. phenylacetica* B4P DSM 14743 and *T. aminoaromatica* S2 DSM 14742 by Deutsche Sammlung von Mikroorganismen und Zellkulturen GmbH (DSMZ) (Braunschweig, Germany). DNA-DNA hybridization studies showed that MZ1T was 100% similar to strain S2, 78.9% to strain B4P and 59.6% to *T. selenatis* ATCC 55363, respectively. When the recommended threshold value of 70% DNA-DNA similarity is used for the definition of bacterial species [4], MZ1T does not belong to the same species as T. selenatis ATCC 55363 but does belong to the same species as strain S2. Based on these results we recommend MZ1T be classified as *Thauera aminoaromatica* strain MZ1T.

**Figure 2:**
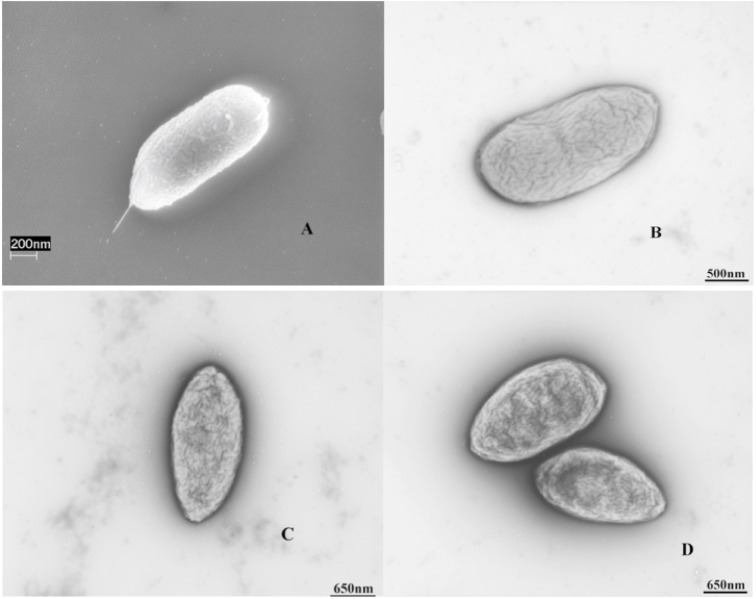
Scanning and transmission electronic microscopic images of *T. aminoaromatica* MZ1T (A and B), S2 (C) and B4P (D).

Morphologically, cells of strain MZ1T are Gram negative, short rods (0.5 x 1.1-1.8 µm) and motile due to the presence of a polar flagellum (Figure 2). Colonies are slimy, creamy white in color at the optimal growth temperature of 30 ºC and pH 7.2, respectively. Strain MZ1T grows aerobically in Stoke’s medium at 30 ºC shaking at 150 rpm and produces copious quantities of extracellular polysaccharide from relatively simple short chain fatty acids at early stationery stage [2]. However, when grown on agar plates, no obvious exopolysaccharide is observed. Under aerobic conditions, benzoate, succinate, aspartate, glutamate, proline, leucine, serine and alanine are utilized. Under anaerobic conditions MZ1T is capable of growth on benzoate with nitrate as the terminal electron acceptor. The characteristic features of the organism are listed in Table 1.

**Table 1:** Classification and general features of *T. aminoaromatica* MZ1T according to the MIGS recommendations [5].

**MIGS ID**	**Property**	**Term**	**Evidence code**
		Domain *Bacteria*	TAS [6]
		Phylum ‘*Proteobacteria*’	TAS [7]]
		Class *Betaproteobacteria*	TAS [8,9]
		Order *Rhodocyclales*	TAS [8,10]
	Current classification	Family *Rhodocyclaceae*	TAS [8,11]
		Genus *Thauera*	TAS [3,12]
		Species *Thauera aminoaromatica*	IDA [3,13,14]
		Strain MZ1T	TAS [1]
	Gram stain	negative	TAS [1]
	Cell shape	rod	TAS [1]
	Motility	motile	TAS [1]
	Sporulation	not reported	
	Temperature range	28-37 ^o^C	TAS [1]
	Optimum temperature	30 ^o^C	TAS [1]
	Salinity	not reported	
MIGS-22	Oxygen requirement	aerobic, facultative	TAS [1]
	Carbon source	numerous 1- and multi-C compounds	TAS [1]
	Energy metabolism	chemolithoautotroph	TAS [1]
MIGS-6	Habitat	fresh water, waste water	TAS [1]
MIGS-15	Biotic relationship	free living	NAS
MIGS-14	Pathogenicity	none	NAS
	Biosafety level	1	TAS [1]
	Isolation	wastewater treatment plant	TAS [1]
MIGS-4	Geographic location	Kingsport, Tennessee, USA	TAS [1]
MIGS-5	Sample collection time	1997	TAS [1]
MIGS-4.1	Latitude	36.548	NAS
MIGS-4.2	Longitude	-82.561	NAS
MIGS-4.3	Depth	NA	
MIGS-4.4	Altitude	369.11 m	NAS

Evidence codes - IDA: Inferred from Direct Assay (first time in publication); TAS: Traceable Author Statement (i.e., a direct report exists in the literature); NAS: Non-traceable Author Statement (i.e., not directly observed for the living, isolated sample, but based on a generally accepted property for the species, or anecdotal evidence). These evidence codes are from the Gene Ontology project [15]. If the evidence code is IDA, the property was directly observed by one of the authors or an expert mentioned in the acknowledgements.

### Chemotaxonomy

The predominant fatty acids found in strain MZ1T are C_16:1 ω7c_ (50.65%), C_16:0_ (25.81%), C_18:1 ω7c_ (9.37%), C_12:0_ (6.3%), C_10:0 3-OH_ (3.87%) and C_12:0 3-OH_ (3.16%). The fatty acid C_12:0 3-OH_ is generally not found in the *Thauera* genus but has been found in *T. selenatis* [12,13]. Therefore, MZ1T is similar to *T. selenatis* based on membrane fatty acid composition.

## Genome sequencing and Annotation

### Genome project history

This organism was selected for sequencing under the DOE Joint Genome Institute (JGI) Community Sequencing Program (CSP). The genome project is deposited in the Genome On Line Database (GOLD) [16] and the complete genome sequence is deposited in GenBank (CP001281). Sequencing, finishing and annotation were performed by the DOE JGI. A summary of the project information is shown in Table 2.

**Table 2:** Genome sequencing project information *T. aminoaromatica* MZ1T.

**MIGS ID**	**Property**	**Term**
MIGS-31	Finishing quality	Finished
MIGS-28	Libraries used	FOSX random whole genome shotgun library
MIGS-29	Sequencing platforms	ABI3730, 454-GS-FLX-Titanium
MIGS-31.2	Sequencing coverage	9.3 × with Sanger, 20 × with 454
MIGS-30	Assemblers	Phrap, Newbler version 2.3
MIGS-34	Gene calling method	Prodigal 1.4, GenePRIMP
	INSDC ID	CP001281 (chromosome) CP001282 (plasmid)
	Genbank Date of Release	August 1, 2009
	GOLD ID	Gc00901
	NCBI project ID	20091
MIGS-13	Source material identifier	MTCC 11151, DSM 25461, LMG 26735
	Project relevance	Bioenergy, Biotechnological, Ecological, Environmental, CSP_776774

### Growth conditions and DNA isolation

Strain MZ1T was grown aerobically in Stoke’s medium at 30 ºC shaking at 150 rpm [2]. Genomic DNA was extracted using a modified Cetyl Trimethyl Ammonium Bromide (CTAB) DNA extraction protocol [17]. Briefly, 100 ml of overnight culture was used for DNA isolation. After incubation with CTAB extraction buffer at 60 ^o^C for 1 hr, cells were lysed and proteins precipitated using an equal volume of chloroform-isoamyl alcohol (24:1), and the aqueous phase was separated, to which one half volume of 5 M NaCl was added followed by two volumes of cold ~ 95% ethanol to precipitate DNA. DNA was dissolved in Tris-EDTA (TE) overnight at (4 to 6 ^o^C). After RNase treatment followed by phenol/chloroform extraction, 1/10 volume of 2 M sodium acetate and 2 volumes absolute ethanol were added to re-precipitate DNA. Finally, DNA was dissolved in TE. The purity, quality and size of the bulk gDNA preparation were assessed by JGI according to DOE-JGI guidelines.

### Genome sequencing and assembly

The genome of *T. aminoaromatica* strain MZ1T was sequenced at the JGI using a combination of 8 kb and 40 kb fosmid DNA libraries. In addition to Sanger sequencing, 454 pyrosequencing was done to a depth of 20 × coverage. All general aspects of library construction and sequencing performed by JGI can be found at the JGI website [18]. Draft assemblies were based on 47,422 total reads. The combined libraries provided 9.0 × coverage. The Phred/Phrap/Consed software package [19] was used for sequence assembly and quality assessment [20-22]. After the shotgun stage, reads were assembled with parallel phrap (High Performance Software, LLC). Possible misassemblies were corrected with Dupfinisher [23] or transposon bombing of bridging clones (Epicentre Biotechnologies, Madison, WI). Gaps between contigs were closed by editing in Consed, custom primer walk or PCR amplification (Roche Applied Science, Indianapolis, IN). A total of 2,230 additional reactions were necessary to close gaps and to raise the quality of the finished sequence. The completed genome sequences of *T. aminoaromatica* strain MZ1T contains 49,771 reads in the chromosome and 2,819 reads in the plasmid, achieving an average of 9.3 × coverage in the chromosome and 29.8 × in the plasmid per base with an error rate 0 in 100,000.

### Genome annotation

The genes were annotated through the Oak Ridge National Laboratory genome annotation pipeline using Prodigal [24] followed by a round of manual curation using the JGI GenePRIMP pipeline [25]. Predicted CDSs were translated and used to search the National Center for Biotechnology Information (NCBI) nonredundant database, UniProt, TIGRFam, Pfam, PRIAM, KEGG, COG, and InterPro databases. Data sources were then combined to assert a product description for each predicted protein. Non-coding genes and miscellaneous features were predicted using tRNAscan-SE [26], RNAMMer [27], Rfam [28], TMHMM [29] and signalP [30].

## Genome properties

The genome contains one chromosome and one plasmid for a total genome size of 4.5 Mb. (Table 3, Figure 3A and Figure 3B). The circular chromosome is 4,496,212 bp in length with a coding density of 89%, a GC content of 68%, 4,071 protein coding genes, 71 structural RNA genes, 93 pseudo genes and 4 copies each of 5S, 16S and 23S rRNA genes. About 62% of predicted genes begin with ATG, 30% begin with TTG, and 7% begin with GTG. Table 4 shows the distribution of genes in COG categories. The plasmid (pTha01) is 78,374 bp in size and has a GC content of 62%, 77% coding density, 75 protein coding genes, 4 pseudo genes and nonstructural RNA genes.

**Table 3:** Genome Statistics for *T. aminoaromatica* strain MZ1T.

**Attribute**	**Value**	**% of Total^a^**
Genome size (bp)	4,574,586	100.00%
DNA coding region (bp)	4,088,809	89.38%
DNA G+C content (bp)	3,124,403	68.30%
Number of replicons	2	
Extrachromosomal elements	1	
Total genes	4,142	100.00%
RNA genes	71	1.71%
rRNA operons	4	
Protein-coding genes	4,071	98.29%
Pseudo genes	93	2.25%
Genes with function prediction	2,980	71.95%
Genes in paralog clusters	2177	52.56%
Genes assigned to COGs	3,163	76.36%
Genes assigned Pfam domains	3330	80.40%
Genes with signal peptides	919	22.19%
Genes with transmembrane helices	976	23.56%
CRISPR repeats	2	

**Figure 3A:**
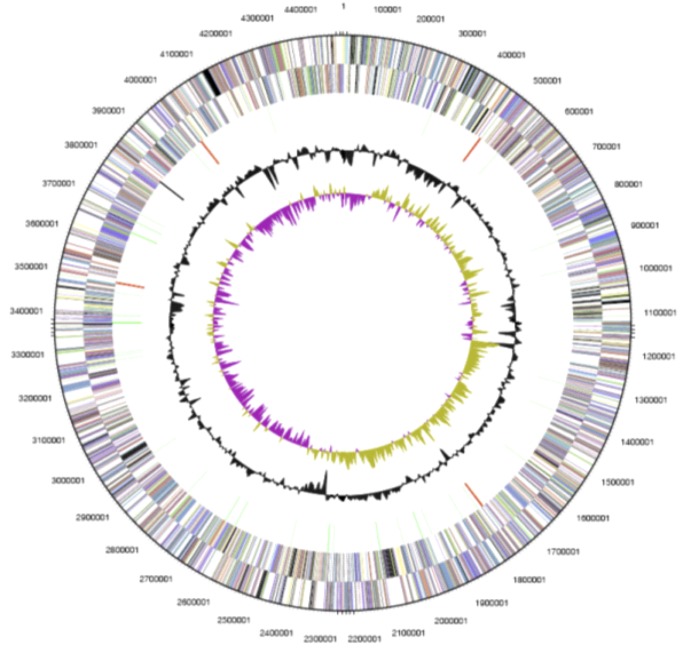
Graphical circular map of the *T. aminoaromatica* MZ1T genome. The outermost two circles (circles 1 and 2) show the genes in the forward and reverse strands, respectively; different colors indicate different function categories. The next circle (circle 3) shows RNA genes (tRNAs green, rRNAs red, other RNAs black); circle 4 shows the GC content, and circle 5 shows the GC skew.

**Figure 3B:**
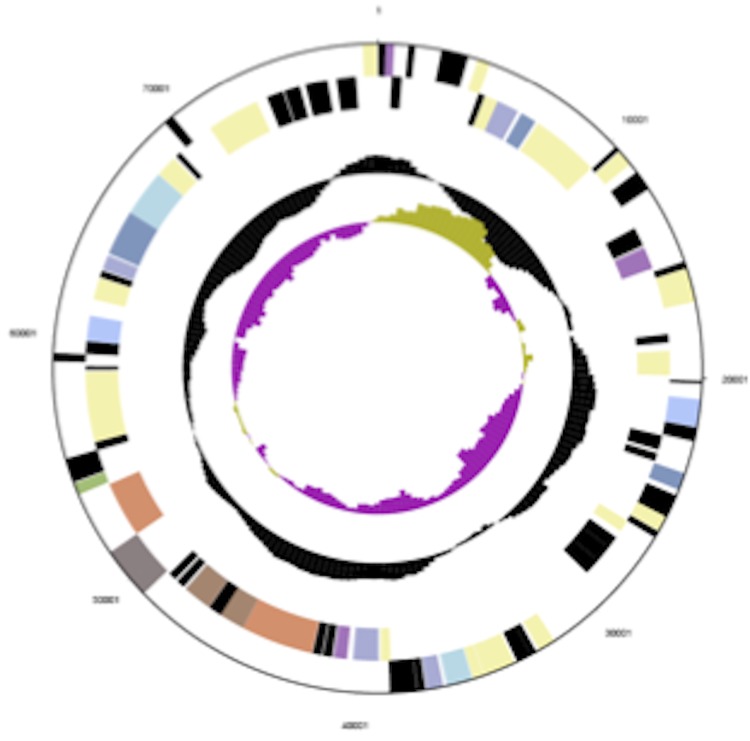
Graphical circular map of the *T. aminoaromatica* MZ1T plasmid pTha01. The outermost two circles (circles 1 and 2) show the genes in the forward and reverse strands, respectively; different colors indicate different function categories. The next circle (circle 3) shows RNA genes (tRNAs green, rRNAs red, other RNAs black); circle 4 shows the GC content, and circle 5 shows the GC skew.

**Table 4:** Number of genes associated with the general COG functional categories

**Code**	**Value**	**% age**	**Description**
J	175	5.01	Translation, ribosomal structure and biogenesis
A	1	0.03	RNA processing and modification
K	215	6.16	Transcription
L	215	6.16	Replication, recombination and repair
B	2	0.06	Chromatin structure and dynamics
D	46	1.32	Cell cycle control, cell division, chromosome partitioning
Y	0	0.0	Nuclear structure
V	68	1.98	Defense mechanisms
T	235	6.73	Signal transduction mechanisms
M	214	6.13	Cell wall/membrane/envelope biogenesis
N	94	2.69	Cell motility
Z	0	0.0	Cytoskeleton
W	0	0.0	Extracellular structures
U	105	3.01	Intracellular trafficking, secretion, and vesicular transport
O	155	4.44	Posttranslational modification, protein turnover, chaperones
C	277	7.93	Energy production and conversion
G	114	3.26	Carbohydrate transport and metabolism
E	276	7.90	Amino acid transport and metabolism
F	73	2.09	Nucleotide transport and metabolism
H	152	4.35	Coenzyme transport and metabolism
I	135	3.87	Lipid transport and metabolism
P	188	5.38	Inorganic ion transport and metabolism
Q	79	2.26	Secondary metabolites biosynthesis, transport and catabolism
R	378	10.82	General function prediction only
S	294	8.42	Function unknown
-	979	23.64	Not in COGs

## Insights from the genome

Annotation of the genome indicated that strain MZ1T has complete glycolytic and citric acid cycle pathways along with two complete acetate assimilation pathways with the key enzymes being acetate-CoA ligase and acetate kinase-phosphate acetyl transferase, respectively, thereby allowing MZ1T to utilize acetate as a carbon source [31]. Three putative gene clusters responsible for exopolysaccharide biosynthesis, polymerization and export were found. The discovery of the *wzy* gene in one of the cluster implicates a Wzy-dependent pathway of polysaccharide synthesis and export in MZ1T [32-34]. Unlike other related *Thauera* spp [35-37], MZ1T does not appear to have genes for anaerobic toluene or phenol degradation; however, genes for both anaerobic and aerobic benzoate degradation are present. The genome of MZ1T contains a total of six sigma factors controlling global gene regulation. These include the housekeeping sigma factor σ^70^, the nitrogen regulator σ^54^, the heat shock sigma factor σ^32^, as well as three copies of *e*xtra*c*ytoplasmic *f*unction (ECF) sigma factor [38]. MZ1T has a large number of genes encoding diverse transporter proteins and those involved in chemotaxis. More than ten copies of two component regulatory systems, genes known to be related to toxin-antitoxin plasmid addiction systems, replication- partition systems and stabilization factors such as Par-like systems were found distributed in both the plasmid and chromosome. Additionally, genes encoding efflux pumps for heavy metal resistance to arsenic, cadmium, lead, silver, zinc but not for selenium have been found on the plasmid. Furthermore, both the plasmid and chromosome contain numerous transposases, integrases and recombinases which demonstrate that genetic rearrangement is widely occurring in this strain.

In liquid culture, MZ1T grows as planktonic cells until late log phase, during which it forms characteristic flocs or cell clusters and then settles out. It was hypothesized that this phenotype may be related to a quorum sensing mechanism. Genes with possible roles in quorum sensing were identified including an acyl-acyl-carrier protein synthase and *lux*R response regulator (12 copies). However, N-acyl-homoserine lactone synthetase or its homologue were not found, which does not support the hypothesis of quorum sensing being one of the mechanisms involved in floc formation. The genome also encodes adhesion related proteins which could be linked to exopolysaccharide production, quorum sensing or “clumping”. Therefore, we speculate that the response of MZ1T to changing environmental conditions involves a complex system involving exopolysaccharide production and flocculation when the cells reach adequate density. Thus, the complete genome sequence of strain MZ1T provides an opportunity to study the biology of important adaptive factors.

## References

[1] Lajoie CA Layton AC Gregory IR Sayler GS Taylor DE Meyers AJ Zoogleal clusters and sludge dewatering potential in an industrial activated-sludge wastewater treatment plant. Water Environ Res 2000; 72:56-64 10.2175/106143000X137112

[2] Allen MS Welch KT Prebyl BS Baker DC Meyers AJ Sayler GS Analysis and glycosyl composition of the exopolysaccharide isolated from the floc-forming wastewater bacterium *Thauera sp.* MZ1T. Environ Microbiol 2004; 6:780-790 10.1111/j.1462-2920.2004.00615.x 15250880

[3] Macy JM Rech S Auling G Dorsch M Stackebrandt E Sly LI *Thauera selenatis* gen. nov., sp. nov., a member of the beta subclass of Proteobacteria with a novel type of anaerobic respiration. Int J Syst Bacteriol 1993; 43:135-142 10.1099/00207713-43-1-135 8427805

[4] Stackebrandt E Frederiksen W Garrity GM Grimont PA Kämpfer P Maiden MC Nesme X Rosselló-Mora R Swings J Trüper HG Report of the ad hoc committee for the re-evaluation of the species definition in bacteriology. Int J Syst Evol Microbiol 2002; 52:1043-1047 10.1099/ijs.0.02360-0 12054223

[5] Field D Garrity G Gray T Morrison N Selengut J Sterk P Tatusova T Thomson N Allen MJ Angiuoli SV The minimum information about a genome sequence (MIGS) specification. Nat Biotechnol 2008; 26:541-547 10.1038/nbt1360 18464787 PMC2409278

[6] Woese CR Kandler O Wheelis ML Towards a natural system of organisms: proposal for the domains Archaea, Bacteria, and Eucarya. Proc Natl Acad Sci USA 1990; 87:4576-4579 10.1073/pnas.87.12.4576 2112744 PMC54159

[7] Garrity GM, Bell JA, Lilburn T. Phylum XIV. Proteobacteria phyl. nov. In: Garrity GM, Brenner DJ, Krieg NR, Staley JT (eds), *Bergey's Manual of Systematic Bacteriology*, second edition, vol. 2 (The Proteobacteria), part B, Springer, New York, 2005, p. 1.

[8] Validation List No 107. List of new names and new combinations previously effectively, but not validly, published. Int J Syst Evol Microbiol 2006; 56:1-6 10.1099/ijs.0.64188-0 16403855

[9] Garrity GM, Bell JA, Lilburn T. Class II. Betaproteobacteria class. nov. In: Garrity GM, Brenner DJ, Krieg NR, Staley JT (eds), *Bergey's Manual of Systematic Bacteriology*, Second Edition, Volume 2, Part C, Springer, New York, 2005, p. 575.

[10] Garrity GM, Bell JA, Lilburn T. Order VI. *Rhodocyclales* ord. nov. In: Garrity GM, Brenner DJ, Krieg NR, Staley JT (eds), *Bergey's Manual of Systematic Bacteriology*, Second Edition, Volume 2, Part C, Springer, New York, 2005, p. 887.

[11] Garrity GM, Bell JA, Lilburn T. Family I. *Rhodocyclaceae* fam. nov., In: DJ Brenner, NR Krieg, JT Staley, (eds) GG (eds), *Bergey's Manual of Systematic Bacteriology*, Second Edition, Volume 2, Part C, Springer, New York, 2005, p. 887.

[12] Heider J, Fuchs G. Genus XI. *Thauera* Macy, Rech, Auling, Dorsch, Stackebrandt and Sly 1993, 139VP emend. Song, Young and Palleroni 1998, 893, In: Garrity G, Brenner DJ, Krieg NR, Staley JR (eds), *Bergey's Manual of Systematic Bacteriology*, Second Edition, Volume 2, Part C, Springer, New York, 2005, p. 907.

[13] Song B Young LY Palleroni NJ Identification of denitrifier strain T1 as *Thauera aromatica* and proposal for emendation of the genus Thauera definition. Int J Syst Syst Bacteriol 1998; 48:889-894 10.1099/00207713-48-3-889 9734042

[14] Anders HJ Kaetzke A Kämper P Ludwig W Fuchs G Taxonomic position of aromatic-degrading denitrifying pseudomonad strains K 172 and KB 740 and their description as new members of the genera *Thauera*, as *Thauera aromatica* sp. nov., and *Azoarcus*, as *Azoarcus evansii* sp. nov., respectively, members of the beta subclass of the Proteobacteria. Int J Syst Bacteriol 1995; 45:327-333 10.1099/00207713-45-2-327 7537067

[15] Ashburner M Ball CA Blake JA Botstein D Butler H Cherry JM Davis AP Dolinski K Dwight SS Eppig JT Gene Ontology: tool for the unification of biology. Nat Genet 2000; 25:25-29 10.1038/75556 10802651 PMC3037419

[16] Liolios K Chen IMA Mavromatis K Tavernarakis N Hugenholtz P Markowitz VM Kyrpides NC The Genomes On Line Database (GOLD) in 2009: status of genomic and metagenomic projects and their associated metadata. Nucleic Acids Res 2010; 38:D346-D354 10.1093/nar/gkp848 19914934 PMC2808860

[17] Porebski S Bailey L Baum B Modification of a CTAB DNA extraction protocol for plants containing high polysaccharide and polyphenol components. Plant Mol Biol Rep 1997; 15:8-15 10.1007/BF02772108

[18] DOE Joint Genome Institute http://www.jgi.doe.gov

[19] Phred/Phrap/Consed software package. http://www.phrap.com

[20] Ewing B Green P Base-calling of automated sequencer traces using phred. I. accuracy assessment. Genome Res 1998; 8:186-194 9521922

[21] Ewing B Hillier L Wendl MC Green P Base-calling of automated sequencer traces using phred. I. accuracy assessment. Genome Res 1998; 8:175-185 9521921 10.1101/gr.8.3.175

[22] Gordon D Abajian C Green P Consed: a graphical tool for sequence finishing. Genome Res 1998; 8:195-202 9521923 10.1101/gr.8.3.195

[23] Han CS, Chain P. Finishing repeat regions automatically with Dupfinisher. In: Arabnia HR, Valafar H (eds). Proceeding of the 2006 international conference on bioinformatics & computational biology. Las Vegas, NV, CSREA Press, 2006, p.141.

[24] Hyatt D Chen GL LoCascio P Land M Larimer F Hauser L Prodigal: prokaryotic gene recognition and translation initiation site identification. BMC Bioinformatics 2010; 11:119 10.1186/1471-2105-11-119 20211023 PMC2848648

[25] Pati A Ivanova NN Mikhailova N Ovchinnikova G Hooper SD Lykidis A Kyrpides NC GenePRIMP: a gene prediction improvement pipeline for prokaryotic genomes. Nat Methods 2010; 7:455-457 10.1038/nmeth.1457 20436475

[26] Lowe TM Eddy SR tRNAscan-SE: a program for improved detection of transfer RNA genes in genomic sequence. Nucleic Acids Res 1997; 25:955-964 9023104 10.1093/nar/25.5.955 PMC146525

[27] Lagesen K Hallin P Rødland EA Stærfeldt HH Rognes T Ussery DW RNAmmer: consistent and rapid annotation of ribosomal RNA genes. Nucleic Acids Res 2007; 35:3100-3108 10.1093/nar/gkm160 17452365 PMC1888812

[28] Griffiths-Jones S Bateman A Marshall M Khanna A Eddy SR Rfam: an RNA family database. Nucleic Acids Res 2003; 31:439-441 10.1093/nar/gkg006 12520045 PMC165453

[29] Krogh A Larsson B von Heijne G Sonnhammer ELL Predicting transmembrane protein topology with a hidden markov model: application to complete genomes. J Mol Biol 2001; 305:567-580 10.1006/jmbi.2000.4315 11152613

[30] Bendtsen DJ Nielsen H von Heijne G Brunak S Improved Prediction of Signal Peptides: SignalP 3.0. J Mol Biol 2004; 340:783-795 10.1016/j.jmb.2004.05.028 15223320

[31] Baldock MI Denger K Smits THM Cook AM Roseovarius sp. strain 217: aerobic taurine dissimilation via acetate kinase and acetate-CoA ligase. FEMS Microbiol Lett 2007; 271:202-206 10.1111/j.1574-6968.2007.00719.x 17425660

[32] Dong C Beis K Nesper J Brunkan-LaMontagne AL Clarke BR Whitfield C Naismith JH Wza the translocon for *E. coli* capsular polysaccharides defines a new class of membrane protein. Nature 2006; 444:226-229 10.1038/nature05267 17086202 PMC3315050

[33] Whitfield C Amor PA Ko¨plin R Modulation of the surface architecture of Gram-negative bacteria by the action of surface polymer:lipid A-core ligase and by determinants of polymer chain length. Mol Microbiol 1997; 23:629-638 10.1046/j.1365-2958.1997.2571614.x 9157235

[34] Whitfield C Roberts IS Structure, assembly and regulation of expression of capsules in *Escherichia coli.* Mol Microbiol 1999; 31:1307-1319 10.1046/j.1365-2958.1999.01276.x 10200953

[35] Evans PJ Mang DT Kim KS Young LY Anaerobic degradation of toluene by a denitrifying bacterium. Appl Environ Microbiol 1991; 57:1139-1145 2059037 10.1128/aem.57.4.1139-1145.1991 PMC182858

[36] Harwood CS Burchhardt G Herrmann H Fuchs G Anaerobic metabolism of aromatic compounds via the benzoyl-CoA pathway. FEMS Microbiol Rev 1998; 22:439-458 10.1111/j.1574-6976.1998.tb00380.x

[37] Shinoda Y Sakai Y Uenishi H Uchihashi Y Hiraishi A Yukawa H Yurimoto H Kato N Aerobic and anaerobic toluene degradation by a newly isolated denitrifying bacterium, *Thauera sp.* Strain DNT-1. Appl Environ Microbiol 2004; 70:1385-1392 10.1128/AEM.70.3.1385-1392.2004 15006757 PMC368410

[38] Gruber TM Gross CA Multiple sigma subunits and the partitioning of bacterial transcription space. Annu Rev Microbiol 2003; 57:441-466 10.1146/annurev.micro.57.030502.090913 14527287

